# The prevalence of medical symptoms in military aircrew

**DOI:** 10.1186/s40696-017-0031-1

**Published:** 2017-02-02

**Authors:** Barak Gordon, Yifat Erlich, Erez Carmon

**Affiliations:** 1 Medical Corps, and Chief Surgeon of the Air Force Headquartes, Israeli Defense Forces, Tel Hashomer, Israel; 2 Sackler Faculty of Medicine, Tel Aviv University, Tel Aviv, Israel

**Keywords:** Symptoms, Aviation medicine, Primary medical care

## Abstract

**Background:**

The prevalence of medical symptoms in aviators has not been described in the medical literature.

**Methods:**

An anonymous questionnaire was handed to all Israeli Air Force aviators who went through the routine yearly examination. Because only two women filled the questionnaire, we excluded them. The questionnaire contained a list of 49 symptoms and the aviators were asked to mark symptoms that were present in the last month before the examination as well as age, estimated weekly flying hours, military service status (reserve or career) and type of aircraft (jet-fighter, helicopter or transport). A general linear model was used to determine the association between age, weekly flying hours, type of aircraft and type of service with the number of symptoms. Binary logistic regression analyses was used to assess the association of these factors with lack of symptoms, and the top five ranking symptoms.

**Results:**

Data was available for 323 male aviators. 62.5% of the aviators reported at least one symptom in the previous month. 26.9% reported three or more symptoms. 25.1% reported spinal symptoms, 22% respiratory symptoms, 21.4% fatigue, 11.5% headache and 6.5% general weakness. Career service was associated with the number of symptoms, fatigue and general weakness. Age was associated with fatigue and general weakness. Aircraft type and weekly flying hours were not associated with any symptom.

**Conclusions:**

Medical symptoms are prevalent in military aviators. Career personnel report on medical symptoms, especially fatigue, more often than reserve personnel. Further study is warranted to examine this association.

## Background

The population of military aviators is unique and consists of usually very fit younger individuals. The selection process to flight academy in the military and periodic medical examinations ensures medical wellbeing. Aviators are exposed to high levels of stress, both physically and mentally, during training and combat. This might put military aircrew in increased risk of developing bodily and psychological symptoms. Although there are studies on the epidemiology of certain complaint or disease in this population [1–4], the issue of subjective symptoms in this population, however, has not been described. It is important to differentiate between symptoms presented to the physician, and the symptoms experienced by the individual [5, 6]. This gap is referred to as the “symptom iceberg.” Some symptoms might disappear with time, some might be self-treated, and some are treated by laypersons (family, neighbors or un-skilled therapists).

Symptoms are associated with poorer perceived health [7], with increased functional impairment and health care use [8, 9], especially with increasing number of symptoms [10]. They could also influence the services a health care system should offer its population, and the practice and education of the primary care physicians in it.

Israeli Air Force (IAF) aviators usually start their training in the flight academy at the age of 18 years. After regular service, they either retire to a very active reserve service, or continue at the military as career military aviators. All aviators (regular, reserve and career) go through yearly medical examination in a single military facility (the IAF Aero-Medical center), and they are required to report any condition that might affect their flight capabilities, although experience has taught us they do not always comply with this regulation. The active service (career and reserve) and the stress it curtails might influence the frequency, severity, types, and number of medical symptoms in this population.

To the best of our knowledge, no research to date has investigated the subjectively experienced medical symptoms in the population of military aviators.

## Methods

All active aviators in the IAF go through mandatory yearly medical examinations. During a single year, beginning on November 26th 2012 a questionnaire was handed to all aviators during their examination. The questionnaire was anonymous and detailed 51 medical symptoms. The aircrew was asked whether he or she experienced any of the symptoms in the last month before the examination. The questionnaire contained personal and demographic, as well as military items. Because the matter of medical symptoms might be very sensitive in active aircrew, and can affect their flight status, the anonymity of the questionnaire was strictly guarded. It was given by hand to the aircrew in the beginning of the examination tour. It contained no identifying marks, and it was emphasized in the introduction to the questionnaire that no identifying marks (such as a personal identity number, name etc.) should be written on it. The questionnaire was filled by the aircrew in his own time, and returned to a sealed container with no human interface. Because only two of the questionnaires were returned by women, they were excluded from this study. Symptoms exclusive to women (such as vaginal pruritus, dysmenorrhea or meno-metrorrhagia) were also excluded from this report.

The study was approved by the Israeli Defense Force Medical Corps institutional review board. Because of the need for strict anonymity formal informed consent was waived. Returning a filled questionnaire was considered as consent.

### Definition of variables

All variables were based on the aircrew self-report. Three types of aircrafts (platforms) were coded: jet-fighters, helicopters and transport fixed wing aircraft. Although a minority of aircrew might fly more than one platform, they usually have a dominant one. No aircrew marked more than one choice in this item. Age was reported in years. Two types of military service were coded: reserve and career service. The mean weekly flight hours in the previous months was divided into three categories: low (0–3), medium (4–6) and high (7 and higher).

### Statistical analysis

Population’s distribution of age, platform, type of service and weekly flying hours and the prevalence of symptoms are descriptive. Two categories of symptoms not present in the questionnaire were added as groups of reported symptoms. Spinal symptoms included all participants reporting on neck, upper or low back pain, and respiratory symptoms included all those reporting on rhinorrhea, cough or dyspnea.

Career personnel are supposedly younger than reserve personnel. A two-tailed t test analysis was performed in order to establish this hypothesis. Collinearity was tested by determining the tolerance and variance inflation factor in a linear regression model.

Univariable binary logistic regression analyses were used to assess the association of age, platform, type of service and weekly flying hours (separately) with no reported symptoms, and the top five symptoms (as rhinorrhea and cough are included in the respiratory symptom and low back pain and neck pain are included in the spinal symptoms category they were not included in the top five most prevalent symptoms for this analysis). As no co-linearity was found (see “Results” section) a multivariable binary logistic model was performed for the symptoms that at least one risk factor was found statistically significant in the univariate model. For the purpose of determining the association of age, platform, type of service and weekly flying hours with the number of symptoms a general linear model was used.

All tests were two-tailed. Statistical significance was considered when P was lower than 0.05. All analyses were performed with the IBM SPSS statistics version 22.

## Results

323 male aviators completed the questionnaire. The mean age (and SD) of participants was 35.93 (10.14) years. Mean age of career aviators was 30.6 (SD 6.65), and the mean age of reserve personnel was 43.34 (SD 8.78). This difference was statistically significant (P < 0.001). No co-linearity was found between all the examined risk factors (variance inflation factor 1.74 for age, and 1.76 for type of service). The distribution of platform, type of service and weekly flying hours of the population is given in Table 1. Most participants were jet-fighter aircrew, most flew low number of hours per week, and almost half were career personnel.

**Table 1:** Population characteristics

Variable	Available			N	Percent of those who answered
	N	Percent			
Aircraft type	308	95.40	Jet fighter	153	49.7
			Helicopter	82	26.6
			Transport	73	23.7
Military type of service	298	92.30	Reserve	132	40.9
			Career	166	51.4
Weekly flying hours	310	96	Low	153	49.4
			Medium	81	26.1
			High	76	24.5

The frequency of symptoms among aviators is presented in Table 2. More than a third reported no symptoms in the previous month, and the top ranking symptoms were spinal symptoms, with low back pain the most frequent of them, respiratory symptoms, with rhinorrhea making the largest part in this category, fatigue (the most prevalent single symptom), headache and general weakness. The number of symptoms reported by each participant is presented in Table 3. More than a third reported one or two symptoms and approximately a quarter reported three or more symptoms.

**Table 2:** Prevalence of symptoms in the previous month

Symptom	N	Percent
No symptoms	121	37.5
Spinal symptoms	81	25.1
Respiratory symptoms	71	22.0
Fatigue	69	21.4
Rhinorrhea	56	17.3
Low back pain	52	16.1
Headache	37	11.5
Cough	34	10.5
Neck pain	28	8.7
Weakness	21	6.5
Thtoat pain	19	5.9
Upper back pain	18	5.6
Knee problems	18	5.6
Nail problems	16	5.0
Toothache	14	4.3
Abdominal distention or gas	14	4.3
Diarrhea	13	4.0
Gum problems	8	2.5
Foot problems	8	2.5
Dizziness	8	2.5
Ankle problems (including sprains)	8	2.5
Anal pain	8	2.5
Visual disturbances	7	2.2
Pruritus	7	2.2
Fever	7	2.2
Allopecia	7	2.2
Abdominal pain	7	2.2
Trauma	6	1.9
Rash	6	1.9
depression	6	1.9
Weight loss	5	1.5
Otalgia	5	1.5
Muscle pain	5	1.5
Eye pain	5	1.5
Upper limb problems	4	1.2
Dyspnea	4	1.2
Palpitations	3	0.9
Hip or thigh pain	3	0.9
Calf pain	3	0.9
Polyuria	2	0.6
Nausea and vomiting	2	0.6
Chills	2	0.6
Chest pain	2	0.6
Sexual dysfunction	1	0.3
Pelvic pain	1	0.3
Hearing disturbances	1	0.3
Breast pain or lump	1	0.3
Anxiety	1	0.3
Dysuria	0	0.0
Disequilibrium	0	0.0
Constipation	0	0.0
Anorexia	0	0.0

**Table 3:** Distribution of the number of symptoms

Number of symptoms	Frequency	Percent
0	121	37.5
1	55	17.0
2	60	18.6
3	32	9.9
4	25	7.7
5	16	5.0
6	7	2.2
7	3	0.9
8	1	0.3
9	1	0.3
10	1	0.3
11	1	0.3
Total	323	100.0

Fatigue and general weakness were significantly associated with a younger age and career service (Table 4). No statistically significant association was observed between the type of aircraft or weekly flying hours and any of the symptoms assessed. After adjustment, odds ratio of age and type of military service still remained statistically significant for fatigue (Odds ratio 0.95, 95% CI 0.91–0.99, P = 0.032, and Odds ratio 2.568, 95% CI 1.104–5.97, P = 0.032, respectively). Odds ratios lost statistical significance in the adjusted model for general weakness. We found a statistically significant association between the type of service and the number of symptoms (career personnel had more symptoms than reservists; P = 0.002). Weekly flying hours, platform and age were not found to be associated with the number of symptoms.

**Table 4:** Risk factors for no-symptoms, and five leading five symptoms in a univariate model

Symptom	Aircraft type			Age	Type of service		Weekly flying hours		
	Jet fighter	Helicopter	Transport	Per year	Reserve	Career	Low	Medium	High
No symptom									
Odds ratio	1.23	1.04	1	1.01	1	0.73	1	0.60	1.16
95% CI	0.69–2.19	0.54–2.01		0.99–1.03		0.46–1.17		0.33–1.07	0.66–2.02
P	0.48	0.9		0.287		0.195		0.083	0.607
Spinal pain									
Odds ratio	1.05	1.05	1	1.01	1	0.73	1	0.96	1.04
95% CI	0.55–1.99	0.51–2.18		0.99–1.04		0.44–1.24		0.51–1.79	0.56–1.95
P	0.893	0.892		0.255		0.247		0.894	0.893
Respiratory									
Odds ratio	0.91	0.74	1	0.98	1	1.67	1	1.80	1.14
95% CI	0.47–1.74	0.35–1.59		0.96–1.01		0.96–2.93		0.96–3.36	0.58–2.26
P	0.768	0.441		0.214		0.071		0.065	0.707
Fatigue									
Odds ratio	1.30	1.10	1	0.93	1	4.31	1	1.15	0.72
95% CI	0.65–2.59	0.50–2.43		0.90–0.96		2.18–8.51		0.61–2.16	0.36–1.47
P	0.462	0.809		<0.001		<0.001		0.670	0.368
Headache									
Odds ratio	0.88	1.39	1	0.98	1	1.07	1	1.53	0.69
95% CI	0.36–2.19	0.54–3.62		0.94–1.01		0.52–2.18		0.70–3.33	0.26–1.82
P	0.788	0.497		0.173		0.855		0.285	0.448
Weakness									
Odds ratio	0.62	0.88	1	0.89	1	16.93	1	1.71	1.28
95% CI	0.21–1.85	0.27–2.86		0.83–0.95		2.24–128.23		0.60–4.91	0.40–4.04
P	0.387	0.834		0.001		0.006		0.315	0.678

## Discussion

In a population of military aviators, we assessed the types and number of medical symptoms experienced in a single month by a questionnaire. Risk factors for the number and most prevalent symptoms were also examined. We found that medical symptoms were experienced by almost two-thirds (62.5%) of the aviators in the previous months, with spinal symptoms and respiratory symptoms leading the list. The most prevalent single symptom reported was fatigue (21.4%), followed by low back pain, rhinorrhea and headache. We also found that younger age and career military service are associated with a higher prevalence of fatigue and general weakness. Career service was also associated with a higher number of symptoms experienced. The type of airplane and the amount of flying hours were not associated with any of the symptoms investigated. To our knowledge, this is the first time that the prevalence of medical symptoms is reported in aviators.

Studies that examined the prevalence of symptoms vary widely in the methods of data acquisition, the population in question, and the definitions of the various symptoms [6]. Data were gathered from questionnaires, or from clinical records. Studies were performed in the general population, or in specific population (such as minority groups). The period of recall varies among studies. This inhibits comparison of our results to previous studies. In studies that collected data on physical symptoms alone, back pain was the most prevalence in some [11], and other musculoskeletal pain in others [12]. In one study that asked for unexplained symptoms, headache was the leading symptom, closely followed by fatigue [5]. Headache, palpitations, nausea and fatigue were the most prevalent in another [13]. Our findings are somewhat different. As single symptoms, fatigue was the most prevalent, followed by low back pain and rhinorrhea. When grouped, back symptoms and respiratory symptoms were most prevalent.

It is interesting to note that a study conducted by Fu and colleagues in Sweden on a civilian aircrew population, reported that eye symptoms were the most prevalent symptoms when the recall period was 7 days (44.6% of the subjects), but when asked to recall symptoms in the last 3 months, tiredness was the most prevalent symptom (64.8%) [14]. In comparison with Fu and colleagues (7 days recall period), the prevalence in symptoms in our population is lower for all similar symptoms: fatigue: (tiredness in Fu and colleagues) 30.9 and 21.4%, respectively; headache 16.9 and 11.5% respectively [14]. We could not be sure of similarity in other symptoms described, but in general, it seems that our population had a lower rate of symptoms. Other studies conducted in the general population also report on higher prevalence of symptoms than we found in our study, although comparison is impossible, as recall time in these studies is usually higher. Al-Windi and colleagues report a prevalence 63% for general fatigue, 51% for backache, and 55% of headache in a Swedish male population aged 25–44 asked to recall symptoms in the last 3 months [15]. In this study the average number of symptoms in a period of 3 months, in males aged 25–44 was 6.6.

The difference in the results in our study compared to previous research probably stems from several reasons. First, the population in our study is very different than in previous ones. The military aviator population are highly selected for both physical fitness and for mental attributes. This population is under stress frequently during flight and other military assignments. It is also younger than the general population. Second, we collected the data on symptom prevalence from questionnaire, and not from medical records. Anonymity was paramount in our study, which might also influence differences in reporting. Third, definitions and understanding of what the meaning of the written symptom in our questionnaire might be different from that in other studies due to the characteristics of the population, and language differences. Fourth, as mentioned above, the recall period of symptoms differs from some studies.

The list of leading causes of medical symptoms in our study warrants adaptation by the healthcare leaders in the IAF and the medical corps of the Israel Defense Forces. Respiratory symptoms are the bread and butter of primary care, but their effect on fitness for flight might be considerable. This necessitates physicians that treat aviators to be proficient in aviation medicine, and to understand the repercussions of even a mild cold on flight status. This is the reason the IAF instructs all the physicians stationed at airbases in aviation medicine. The propensity of back and neck symptoms points to the need of available orthopedic and supportive services (such as physiotherapy, chiropracy, osteopathy). This process has been applied in previous years, and intensified recently in the IAF.

We also found that career service, in comparison to reserve service is associated with increased risk for the prevalence of fatigue and general weakness, although in the case of general weakness we could not establish that the increased risk for career servicemen was independent of their younger age. We also found that career personnel reported more symptoms that reservists. These findings possibly originate from the intensity of this type of service, and the protective effect of the diversity in occupations of reserve aircrew. The prevalence of fatigue and general weakness might be associated with burn-out, depressive symptoms, or anxiety [16, 17]. The IAF has a dedicated psychology clinic for aviators, and a high rate of anxiety or depression among career personnel has not been clinically observed. In the wake of the current research, the psychology branch intends to conduct a study to examine these concerns.

The findings that weekly flying hours and type of plane were not associated with any symptoms nor with the total number of symptoms possibly mean that flight itself is not the trigger. As described in a previous study [18], the prevalence of medical symptoms as a whole in our population did not differ significantly from that in general populations as described in various studies. There is, however, a wide range of symptoms’ prevalence described. These probably stem from fundamental variance in data gathering methods in these studies. In order to differentiate symptoms associated with flight from “background” prevalence of symptoms (defined as the prevalence in a non-flyer age-matched population) targeted studies are warranted, and these are outside the scope of the current study.

There are some limitations to our study. The study was based on a self-filled questionnaire. This might lead to a bias due to a fear from repercussions if symptoms were raised, such as loosing flight status, further tests etc. We believe that strictly guarding the anonymity of the aviator, as previously described, minimizes this concern. Furthermore, in a previous study we found that most aviators that do not report their symptoms to the physician do so not because of fear of repercussions, but because of other reasons (such as not deeming the symptoms as unimportant, or lack of time to investigate it) [18]. We asked on symptoms during the previous month. This might lead to a recall bias. We tried to minimize its effect by detailing all the symptoms in the questionnaire. Another possible bias is a selective response bias, meaning that those with symptoms might tend to fill the questionnaire more than those not suffering from symptoms. This might lead to overestimation of symptom prevalence.

## Conclusions

Medical symptoms are prevalent in military aviators, although probably not in severe forms. The most prevalent are spinal complaints, respiratory symptoms and fatigue. Career service is associated both with a higher number of symptoms and with fatigue and general weakness, while flying hours and type of aircraft do not seem to influence the prevalence of symptoms. Further research is warranted as to the reasons for higher symptom burden in career aviators. Studies are needed in order to see symptom patterns in other military and aircrew populations.
